# Robotic versus laparoscopic right hemicolectomy: a case-matched study

**DOI:** 10.1007/s11701-021-01286-5

**Published:** 2021-08-02

**Authors:** Enda Hannan, Gerard Feeney, Mohammad Fahad Ullah, Claire Ryan, Emma McNamara, David Waldron, Eoghan Condon, John Calvin Coffey, Colin Peirce

**Affiliations:** 1 Department of Colorectal Surgery, University Hospital Limerick, St Nessan’s Road, Dooradoyle Co, Limerick, Ireland; 2 School of Medicine, University of Limerick, Limerick, Ireland

**Keywords:** Robotic surgery, Laparoscopic surgery, Colon cancer, Right hemicolectomy

## Abstract

The current gold standard surgical treatment for right colonic malignancy is the laparoscopic right hemicolectomy (LRH). However, laparoscopic surgery has limitations which can be overcome by robotic surgery. The benefits of robotics for rectal cancer are widely accepted but its use for right hemicolectomy remains controversial. The aim of this study was to compare outcomes in patients undergoing robotic right hemicolectomy (RRH) and LRH in a university teaching hospital. Demographic, perioperative and postoperative data along with early oncological outcomes of patients who underwent RRH and LRH with extracorporeal anastomosis (ECA) were identified from a prospectively maintained database. A total of 70 patients (35 RRH, 35 LRH) were identified over a 4-year period. No statistically significant differences in estimated blood loss, conversion to open surgery, postoperative complications, anastomotic leak, 30-day reoperation, 30-day mortality, surgical site infection or lengths of stay were demonstrated. Surgical specimen quality in both groups was favourable. The mean duration of surgery was longer in RRH (*p* <  < 0.00001). A statistically significant proportion of RRH patients had a higher BMI and ASA grade. The results demonstrate that RRH is safe and feasible when compared to LRH, with no statistical difference in postoperative morbidity, mortality and early oncological outcomes. A difference was noted in operating time, however was influenced by training residents in docking the robot and a technically challenging cohort of patients. Operative time has shortened with further experience. Incorporating an intracorporeal anastomosis technique in RRH offers the potential to improve outcomes compared to LRH.

## Introduction

Minimally invasive surgery is recognised as the gold standard of treatment for colon cancer due to well-demonstrated benefits in terms of 30-day post-operative outcomes and equivalent long-term oncological results compared to open approaches in numerous multicentre randomised controlled trials [1]. Minimally invasive approaches to the right colon have been well described, with several studies showing that such techniques provide numerous advantages to open surgery that include lower intra-operative blood loss, less post-operative pain, faster recovery of intestinal function, a shorter hospital stay and earlier recovery of normal activity [2]. Currently, right hemicolectomy for cancer is predominantly performed in high volume centres via a laparoscopic approach which has been demonstrated to be safe and feasible [3]. However, laparoscopic approaches are not without limitations. These include an assistant-dependent unstable two-dimensional view, exaggerated tremor, limited ergonomics, the difficulty in performing high precision suturing and the diminished dexterity offered by rigid instruments with fixed tips compared to that of the surgeon’s hands [4]. Furthermore, it has been shown that laparoscopic right hemicolectomy (LRH) has a significantly steeper learning curve than the open approach, and thus obtaining a proficiency in the technique can be challenging for trainee surgeons [5].

Robotic surgical platforms such as the da Vinci surgical system have been designed to address the many limitations of laparoscopic surgery. This is achieved by offering a stable three-dimensional view that is directly controlled by the operating surgeon, tremor-eliminating technology, improved ergonomics and a greater instrument range of motion with 7 degrees of freedom, 180° articulation and 540° rotation [6]. Furthermore, it has been shown that the learning curve for robotic surgery is less steep compared to laparoscopic techniques [7]. Such advantages have made robotic surgery attractive to colorectal surgeons, particularly in the realm of rectal surgery, where the improved optics and dexterity offered in the highly challenging and potentially hazardous operative field of the narrow bony pelvis have proven beneficial [8, 9]. However, robotic surgery has faced criticism due to an association with higher cost due to the inherent expense and maintenance of robotic platforms, along with a longer time in the operating theatre due to the process of setting up and docking the robot [10].

While the benefits of robotic surgical platforms over laparoscopic approaches in the context of rectal surgery have been demonstrated, it remains controversial as to whether or not such an approach offers any advantage with regards to right hemicolectomy [11]. A common criticism of robotic right hemicolectomy (RRH), along with increased cost and longer operating time, is that the inherent advantages of robotic platforms are less pronounced in the more spacious intra-abdominal cavity than in the pelvis, with clashing of instruments due to inhibited ergonomics and impaired range of motion being far less burdensome than in rectal surgery [11]. For these reasons, RRH has not yet fully penetrated the practice of colorectal surgery [12]. However, the robotic characteristics, including improved vision, ergonomics and tremor filtering, may translate to more precise and fine dissection compared to laparoscopy [13]. Currently, there are few studies comparing outcomes between RRH and LRH, which has drawn less interest than such comparisons in rectal surgery [14]. The purpose of this study was to compare the outcomes of RRH with LRH in our institution, a tertiary referral university teaching hospital with a robotic colorectal surgery programme established in 2016 and a laparoscopic colorectal surgery programme in 2008.

## Methods

### Patient selection and data collection

A retrospective analysis of a prospectively maintained database was conducted for this study. The database comprised all patients who underwent robotic colorectal surgery in our institution since its introduction in 2016. All patients that underwent a RRH during this time period for both benign and malignant pathology were included. These were case-matched with an equivalent number of patients that underwent LRH during the same time period which were identified from the operating theatre logbooks. Procedures that deviated from oncological principles without high ligation of the ileocolic artery, such as ileocolic resections and caecectomies, were excluded from the study. Extended right hemicolectomies were also excluded from the study. Demographic, perioperative, postoperative and surgical specimen data were collected from the database. In cases where the required data were not available from the database, a review of patient medical records was performed. All cancer patients were discussed at the colorectal multidisciplinary meeting (MDT) prior to operative intervention. Ethical approval was granted by the hospital research ethics committee. All operations were performed by four fellowship-trained consultant colorectal surgeons on the specialist division of the medical register. Those that performed RRH had completed proctorship programmes in robotic colorectal surgery.

### RRH surgical technique

All operations were performed using the da Vinci^®^ Xi dual console robotic surgical system (Intuitive Surgical Inc, Sunnyvale, CA, USA). With the patient under general anaesthesia and in the supine position, pneumoperitoneum was established either via an 8 mm robotic port placed subumbilical by Hasson technique. Placement of three further 8 mm robotic trocars followed the recommended manufacturer guidelines so that the four trocars were positioned in a line perpendicular to the target anatomy at a range 6–10 cm apart, extending from 4 cm above the pubic symphysis to the left subcostal margin in the midclavicular line, with a further 12 mm assistant port placed as far a distance as possible from the da Vinci^®^ ports and lateral to the left midclavicular line. A medial-to-lateral approach to dissection was performed with a high ligation of the ileocolic pedicle performed using the Weck^®^ Hem-o-lock^®^ clips. Following this, mobilisation of the right colon was completed in a lateral-to-medial manner, with the hepatic flexure mobilised. Following completion of mobilisation, the subumbilical incision was extended in the midline to allow exteriorisation of the specimen, where resection was completed following division of the mesentery ensuring appropriate resection margins. A combination of stapled and handsewn techniques for extracorporeal anastomosis (ECA) with or without omentopexy were used based on surgeon preference.

### LRH surgical technique

With the patient under general anaesthesia and in the supine position, pneumoperitoneum was established either via a 12 mm subumbilical port placed by Hasson technique or by insertion of a Veress needle based on surgeon preference. Three 5 mm trocars were placed under laparoscopic vision, with two being used by the operating surgeon and one being used by the assistant to provide retraction. Exact port position varied according to patient body habitus and surgeon preference. A medial to lateral approach to dissection was performed with the ileocolic pedicle divided using the Ligasure™ 5 mm blunt tip laparoscopic instrument, with mobilisation of the right colon completed in a similar manner to the technique used in RRH. Specimen exteriorisation, resection and extracorporeal ileocolic anastomosis was also completed via extension of the subumbilical incision in the midline, with ECA technique based on surgeon preference.

### Statistical analysis

Statistical analysis was performed using IBM SPSS version 24 (SPSS Inc, Chicago, IL, USA). Non-parametric data were expressed as median with interquartile range and parametric data as a mean with standard deviation. Univariate analysis was performed using a Student’s *t* test or Mann Whitney *U* test for continuous variables, and a Fischer’s exact test for categorical variables. A *p* value of < 0.05 was considered statistically significant.

## Results

### Baseline characteristics

Between July 2016 and July 2020, 35 patients underwent RRH, which were case-matched to 35 patients that underwent LRH during the same time period in our institution which were randomly selected from the operating theatre logbooks to avoid selection bias. The mean age of the included 70 patients was 67.8 years (range 31–90 years) and the majority (51.4%, *n* = 36) were male. The indication for surgery in most cases was for malignancy (60%, *n* = 42). The majority (52.9%, *n* = 37) had an American Society of Anaesthesiologists (ASA) grade of 2. Twenty two patients (31.4%) had a body mass index that classified them as obese (BMI ≥ 30.0 kg/m^2^). Patient baseline characteristics were comparable between the two groups apart from BMI, where a higher proportion of patients in the RRH cohort (48.6%) suffered with obesity compared to the LRH cohort (14.2%) (*p* = 0.001) (Table 1).

**Table 1:** Baseline characteristics

	Overall (*n* = 70)	RRH (*n* = 35)	LRH (*n* = 35)	*p* value
Mean age (years)	67.8	66.5	69.7	0.16
Gender				
Male	36 (51.4%)	18 (51.4%)	18 (51.4%)	1.0
Female	34 (48.6%)	17 (48.6%)	17 (48.6%)	1.0
ASA grade				
I	21 (30%)	7 (20%)	14 (40%)	**0.03**
II	37 (52.9%)	20 (57.1%)	17 (48.6%)	0.24
III	12 (17.1%)	8 (22.9%)	4 (11.4%)	0.1
BMI				
≥ 30.0 kg/m^2^	22 (31.4%)	17 (48.6%)	5 (14.2%)	**0.001**
< 30 kg/m^2^	48 (68.6%)	18 (51.4%)	30 (85.8%)	**0.001**
Diagnosis				
Malignancy	48 (68.6%)	20 (57.1%)	28 (80%)	**0.02**
Benign polyp	17 (24.3%)	10 (28.6%)	7 (20%)	0.2
Crohn’s disease	5 (7.1%)	5 (14.3%)	0 (0%)	**0.01**
Pathological T Stage (*n* = 48)				
T1	10 (20.8%)	5 (25%)	5 (17.9%)	0.27
T2	9 (18.8%)	1 (5%)	8 (28.6%)	**0.02**
T3	22 (45.8%)	11 (55%)	11 (39.3%)	0.14
T4	7 (14.6%)	3 (15%)	4 (14.2%)	0.47

*p* values less than 0.05 was deemed statistically significant are highlighted in bold

### Perioperative outcomes

The median duration of surgery was 216 min in the RRH cohort and 105 min in the LRH cohort. The duration of surgery in the RRH group was inclusive of a median time of 32 min from commencing surgery to docking the robot. The median duration of surgery for RRH in the first year of the programme was 269.5 min compared to 190 min in the most recent year. The mean intra-operative blood loss was 110 ml in the RRH cohort and 92 ml in the LRH cohort. The rate of conversion to open surgery was 2.9% (*n* = 1) in the RRH cohort and 0% in the LRH cohort. Conversion to open surgery was defined as an unplanned midline laparotomy due to inability to complete a planned robotic or laparoscopic stage of the operation. One patient had a loop ileostomy formed in the LRH group, resulting in a stoma rate of 2.9% (*n* = 1) while no patients required stoma formation at their index operation in the RRH group (Table 2).

**Table 2:** Perioperative outcomes

	RRH (*n* = 35)	LRH (*n* = 35)	*p* value
Median duration of surgery (minutes)	216 (32 min docking)	105	** < 0.00001**
Median estimated blood loss (ml)	110	92	0.14
Conversion to open surgery (%)	2.9% (*n* = 1)	0% (*n* = 0)	0.16
Stoma formation (%)	0%	2.9%	0.16

*p* values less than 0.05 was deemed statistically significant are highlighted in bold

### Postoperative outcomes

The median length of stay was 6 days (range 3–66) in those that underwent RRH and 6 days (range 4–24) in those that underwent LRH. The surgical site infection (SSI) rate was 11.4% (*n* = 4) in patients that underwent RRH compared to 14.3% (*n *− 5) in those managed by LRH (*p* = 0.36). One anastomotic leak was recorded in patients that underwent RRH (2.9%), while none were recorded in the LRH cohort. One patient in the RRH group required reoperation within 30 days of surgery, which was to manage an anastomotic leak by resection of the anastomosis and end ileostomy formation. The 30-day reoperation rate in the LRH group was 0%. Two patients (5.8%) in the LRH cohort developed postoperative subhepatic collections requiring drainage by interventional radiology. No 30-day mortalities were recorded in either group (Table 3).

**Table 3:** Postoperative outcomes and surgical specimen quality

	RRH (*n* = 35)	LRH (*n* = 35)	*p* value
Median length of stay (days)	6	6	0.25
Postoperative complication (%)	17.1% (*n* = 6)	22.9% (*n* = 8)	0.27
Anastomotic leak (%)	2.9% (*n* = 1)	0% (*n* = 0)	0.16
30-day reoperation (%)	2.9% (*n* = 1)	0% (*n* = 0)	0.16
30-day mortality (%)	0% (*n* = 0)	0% (*n* = 0)	1.0
SSI (%)	11.4% (*n* = 4)	14.3% (*n* = 5)	0.36
Median lymph node yield	17.5	19	0.11
R1 margins (%)	0% (*n* = 0)	0% (*n* = 0)	1.0

### Surgical specimen quality

The median lymph node yield was 19 lymph nodes in the LRH cohort and 17.5 in the RRH cohort, and no patient had a positive margin (Table 3).

## Discussion

The present study analysed 70 minimally invasive right hemicolectomies (35 RRH versus 35 LRH) performed in our institution, with the results demonstrating that the two approaches are comparable in terms of postoperative outcomes and surgical specimen quality. Perioperative outcomes were also equivalent except for a statistically significant difference in median duration of surgery, however this longer operating time did not result in a higher postoperative complication incidence in the RRH group. These results demonstrate that RRH can be regarded as a safe and feasible procedure which is non-inferior to LRH.

Debate and speculation exist regarding the routine use of robotics in right-sided colonic surgery [15]. The main criticisms aimed at RRH are longer operating times, higher costs and loss of haptic feedback compared with a laparoscopic approach [15]. In the current study, it was observed that RRH was associated with a longer operative time than LRH. Increased duration of surgery in RRH is influenced by a variety of factors such as learning curve, set-up times and cart docking times [14–16]. However, it is the authors’ experience that with increased experience and caseload, the docking times become more efficient [8, 9]. It is important to highlight that, in the current study, cases from a long-established laparoscopic surgery programme have been compared to the initial 35 cases from a new robotic surgery programme. During the study period, both the median duration of surgery and median docking time for RRH trended downwards while operating times for LRH remained stable during the same period. It would be expected that operating times for RRH would continue to improve as experience increases, while those for LRH will likely remain unchanged. Other institutions have reported improvements in operating time for RRH with greater caseload and the same is expected in our institution [15].

Other factors may have also contributed to the longer operative time observed in RRH compared to LRH. Firstly, a significant proportion of patients managed by RRH were obese compared with that of the LRH cohort (*p* = 0.001). Obesity poses many challenges in minimally invasive colorectal surgery and is associated with increased perioperative and postoperative complications, longer operating times, longer lengths of stay and increased healthcare expenditure [17]. It is also a specific risk factor for SSI, wound dehiscence, incisional hernia and anastomotic leak [17, 18]. It has been demonstrated that obese patients pose a greater technical challenge for minimally invasive colorectal surgery as they tend to have a thicker mesentery and larger omentum, which can result in impaired vision, restricted space for instruments to manoeuvre, distorted surgical planes and troublesome bleeding [17, 18]. For these reasons, the RRH cohort likely posed a greater technical challenge than the less obese LRH cohort, which may have contributed to the longer operating time in patients managed by robotic surgery [18–20]. However, it is interesting to note that, despite the potential greater technical challenge posed by those undergoing RRH, the well-recognised higher complication rate associated with obese patients was not observed, with equivalent post-operative outcomes to those that underwent LRH. The findings that the more obese, and thus, potentially more technically challenging RRH cohort had non-inferior outcomes to the less obese LRH cohort is favourable for a robotic approach.

Secondly, the RRH cohort may have also posed a greater intraoperative challenge given that a statistically significant proportion of patients underwent surgery for Crohn’s disease compared to the LRH cohort (*p* = 0.01), which is well recognised as one of the most challenging pathologies to manage operatively in colorectal surgery, and thus may have impacted the operating time [5]. Thirdly, patients that underwent RRH also were largely more comorbid than the LRH group, with a higher proportion of patients in the LRH cohort having an ASA grade of I compared to RRH (0 = 0.03). Despite this, these patients did not require a longer inpatient length of stay and did not have a higher complication rate.

Finally, the longer operating time in the RRH group may also be accounted for by the focus on training and education within the robotic surgery programme. Particular time and emphasis is placed on ensuring that trainees learn the principles of appropriate port setup and robotic docking that are essential for performing safe robotic surgery, and are encouraged to perform these steps under supervision to ensure competency, and the consultant usually remains unscrubbed for this. It is inevitable that this would lead to a longer docking time than if these steps were performed primarily by a consultant. Similarly, trainees in our institution also benefit greatly from the dual console system and are offered an opportunity to perform various steps of the procedure under consultant supervision after completion of the relevant training modules, which also contributes to the longer operating time. A similar emphasis on training is offered in the laparoscopic surgery programme in our institution, however most colorectal trainees would have previous experience with laparoscopic colorectal surgery and thus this would be less likely to impact operating time to the same degree as in robotic surgery. The RRH offers an important opportunity for residents to train in robotic surgery [12]. While it is possible that this emphasis on training for residents in the robotic surgery programme may contribute to longer operating times, it is important to note that this focus on surgical education does not appear to have had a detrimental impact on postoperative outcomes, suggesting that such inclusion of trainees in robotic colorectal surgery is safe and feasible. This is supported by a recent publication by Collins et al. where no differences in intraoperative or postoperative complications were observed in robotic rectal surgery performed by fellows compared with cases performed by consultants [21].

It is important to emphasise that this study reports on the first 35 cases of RRH performed in our centre, which already compare favourably to a well-established laparoscopic practice. International literature suggests that the learning curve for RRH is complete after 45 procedures, at which point the technical skills necessary to significantly reduce operative time, conversion to open surgery and to significantly improve the number of harvested lymph nodes are obtained [21]. Even at this relatively early stage of our experience with robotic surgery, outcomes that are equivalent to those of LRH have been observed in all domains except for operating time, with a strong evidence basis showing that efficiency in RRH can be expected to significantly improve with increased experience [22]. Nonetheless, there exists significant potential to improve. In all cases included in this study, an ECA was performed. However, a specific advantage that RRH offers is the potential to perform an intracorporeal anastomosis (ICA). Standard international practice for LRH is to exteriorise the bowel through a mini-laparotomy and to perform an ECA, with most surgeons being uncomfortable with performing laparoscopic ICA due to technical challenge and the lack of stability afforded by laparoscopic instruments [23]. However, the inherent characteristics of robotic surgical platforms allow for ICA to be performed with a stability and precision that cannot be achieved laparoscopically, and therefore is becoming increasingly common practice in RRH [15]. This offers many advantages, with a mini-laparotomy incision no longer being necessary, resulting in a shorter length of stay, less post-operative pain, better cosmetic outcomes and less wound complications such as SSI, fascial dehiscence or incisional hernia [15]. ICA also allows direct visualization of the mesentery at the time of anastomosis, which can help prevent a twist on the mesentery [23]. It is likely that ICA may initially further increase operative times, however, these will then reduce as the learning curve progresses, and may be justified by the positive impact on post-operative morbidity and length of stay [15]. These widely reported favourable experiences with ICA show that there is further potential for our outcomes in RRH to improve by including this in our surgical armamentarium, while the well-established laparoscopic technique with ECA has likely reached its peak potential [15].

The current study is not without limitations. The study was conducted in a single centre, was retrospective in nature and focused on a small number of patients. Nonetheless, our results demonstrate that RRH is safe and feasible when compared to LRH, with no statistical difference in regards to postoperative morbidity, mortality and surgical specimen quality. RRH offers an invaluable training opportunity for colorectal trainees to gain experience and confidence in robotic surgery, allowing them to acquire the skills necessary to safely progress to more complex parts of the dissection and further operations. This study compares the first cases performed in a robotic surgery programme with a well-established laparoscopic surgery programme, with favourable outcomes even at this early stage. It has been observed that the operative time has shortened with further experience, and this is expected to continue to improve with an increased caseload. Incorporating an ICA technique offers the potential to improve our outcomes compared to LRH.

## Data Availability

The data used for this study are available on reasonable written request to the authors.

## Abbreviations

RRH: Robotic right hemicolectomy
LRH: Laparoscopic right hemicolectomy
BMI: Body mass index
ASA: American Society of Anaesthesiologists
ECA: Extracorporeal anastomosis
ICA: Intracorporeal anastomosis

## Code availability

Statistical analysis was performed using IBM SPSS version 24 (SPSS Inc, Chicago, IL, USA).

## References

[1] Formisano G, Misitano P, Giuliani G (2016). Laparoscopic versus robotic right colectomy: technique and outcomes. Updates Surg.

[2] Genova P, Pantuso G, Cipolla C (2020). Laparoscopic versus robotic right colectomy with extra-corporeal or intra-corporeal anastomosis: a systematic review and meta-analysis. Langenbecks Arch Surg.

[3] Crawshaw BP, Chien H, Augestad KM (2015). Effect of laparoscopic surgery on health care utilization and costs in patients who undergo colectomy. JAMA Surg.

[4] Park JS, Choi G-S, Lim KH, Jang YS, Jun SH (2011). S052: a comparison of robot-assisted, laparoscopic, and open surgery in the treatment of rectal cancer. Surg Endosc.

[5] Reissman P, Cohen S, Weiss EG, Wexner SD (1996). Laparoscopic colorectal surgery: ascending the learning curve. World J Surg.

[6] Tan A, Ashrafian H, Scott AJ (2016). Robotic surgery: disruptive innovation or unfulfilled promise? A systematic review and meta-analysis of the first 30 years. Surg Endosc.

[7] Baek SK, Carmichael JC, Pigazzi A (2013). Robotic surgery: colon and rectum. Cancer J.

[8] Fleming CA, Westby D, Ullah MF (2020). A review of clinical and oncological outcomes following the introduction of the first robotic colorectal surgery programme to a university teaching hospital in Ireland using a dual console training platform. J Robot Surg.

[9] Fleming CA, Ullah MF, Chang KH (2020). Propensity score-matched analysis comparing laparoscopic to robotic surgery for colorectal cancer shows comparable clinical and oncological outcomes. J Robot Surg.

[10] Jayne D, Pigazzi A, Marshall H, Croft J (2017). Effect of robotic-assisted vs conventional laparoscopic surgery on risk of conversion to open laparotomy among patients undergoing resection for rectal cancer: the ROLARR randomized clinical trial. JAMA.

[11] Pigazzi A, Garcia-Aguilar J (2010). Robotic colorectal surgery: for whom and for what?. Dis Colon Rectum.

[12] deSouza AL, Prasad LM, Park JJ, Marecik SJ, Blumetti J, Abcarian H (2010). Robotic assistance in right hemicolectomy: is there a role?. Dis Colon Rectum.

[13] Spinoglio G, Bianchi PP, Marano A (2018). Robotic versus laparoscopic right colectomy with complete mesocolic excision for the treatment of colon cancer: perioperative outcomes and 5-year survival in a consecutive series of 202 patients. Ann Surg Oncol.

[14] Xu H, Li J, Sun Y (2014). Robotic versus laparoscopic right colectomy: a meta-analysis. World J Surg Oncol.

[15] Waters PS, Cheung FP, Peacock O (2020). Successful patient-orientated surgical outcomes in robotic vs laparoscopic right hemicolectomy for cancer: a systematic review. Colorectal Dis.

[16] Kim HJ, Choi GS, Park JS (2014). Multidimensional analysis of the learning curve for robotic total mesorectal excision for rectal cancer: lessons from a single surgeon’s experience. Dis Colon Rectum.

[17] Pikarsky AJ, Saida Y, Yamaguchi T (2002). Is obesity a high-risk factor for laparoscopic colorectal surgery?. Surg Endosc.

[18] Lascano C, Kaidar-Person O, Szomstein S (2006). Challenges of laparoscopic colectomy in the obese patient: a review. Am J Surg.

[19] Fleshman J, Sargent DJ, Green E (2007). Clinical outcomes of surgical therapy study group. Laparoscopic colectomy for cancer is not inferior to open surgery based on 5 year data from the COST study group trial. Ann Surg.

[20] Suwa Y, Joshi M, Poynter L (2020). Obese patients and robotic colorectal surgery: systematic review and meta-analysis. BJS Open.

[21] Collins D, Machairas N, Duchalais E (2018). Participation of colon and rectal fellows in robotic rectal cancer surgery: effect on surgical outcomes. J Surg Educ.

[22] Parisi A, Scrucca L, Desiderio J, Gemini A, Guarino S, Ricci F, Cirocchi R, Palazzini G, D'Andrea V, Minelli L, Trastulli S (2017). Robotic right hemicolectomy: analysis of 108 consecutive procedures and multidimensional assessment of the learning curve. Surg Oncol.

[23] Morpurgo E, Contardo T, Molaro R (2013). Robotic-assisted intracorporeal anastomosis versus extracorporeal anastomosis in laparoscopic right hemicolectomy for cancer: a case control study. J Laparoendosc Adv Surg Tech A.

